# Isolation and Characterization of Lipase-Producing Bacteria in the Intestine of the Silkworm, *Bombyx mori*, Reared on Different Forage

**DOI:** 10.1673/031.011.13501

**Published:** 2011-10-07

**Authors:** Wei Feng, Xiao-Qiang Wang, Wei Zhou, Guang-Ying Liu, Yong-Ji Wan

**Affiliations:** Laboratory of Invertebrate Pathology and Applied Microbiology, College of Biotechnology, Southwest University, Chongqing 400716, China

**Keywords:** Lepidoptera, dominant bacterium, lipase activity, diversity, mulberry leaf, tricuspid cudrania leaf

## Abstract

The silkworm, *Bombyx mori* L. (Lepidoptera: Bombycidae), an oligophagous insect that mainly feeds on mulberry leaves, is susceptible to entomopathogen infection when reared with tricuspid cudrania leaves. A total of 56 dominant bacterial strains, classified into 12 phylotypes based on bacteriological properties and analysis of 16S rRNA genes, were isolated from the intestine of the fourth and fifth instar silkworm larvae. Ten and seven phylotypes exist in the intestine of the silkworm larvae reared with mulberry leaves and tricuspid cudrania leaves, respectively. Four of them are common in the intestine of the two treatment groups. By screening their lipolytic ability on a Rhodamine B agar plate, nine lipase-producing bacterial strains were obtained and classified into six genera, including *Bacillus*, *Brevibacterium*, *Corynebacterium*, *Staphylococcus*, *Klebsiella*, and *Stenotrophomonas.* Except for *Stenotrophomonas*, which is common in both, the other genera only exist in the intestine of the silkworm larvae fed with mulberry leaves. In addition, by culture and fermentation *in vitro*, the maximum cell density and lipase activity of lipase-producing bacteria were examined at about 48 hours. The results indicate that diet has a significant impact on the gut bacterial community, especially lipase-producing bacteria. We suggest that the difference of lipase-producing bacterial diversity might be related to disease resistance of the silkworm.

## Introduction

Insect guts harbor an abundance of microorganisms, which play important roles in the host's nutrition, development, resistance to pathogens, and reproduction (Brand et al. 1975; Brune 2003; Moran et al. 2005). Loss of microorganisms often results in abnormal development and reduces survival of the insect host (Eutick et al. 1978; Fukatsu and Hosokawa 2002). Compared to mammals, there is a lack of literature regarding the functions of microorganisms in insect guts. Several species, such as termites and desert locusts, are the only insects whose gut microorganisms have been extensively studied (Dillon and Charnley 2002; Ohkuma 2003; Hongoh et al. 2005). Some specific roles of microorganisms in these insect guts have been disclosed, including lignocellulose digestion, methanogenesis, acetogenesis from H_2_ and CO_2_, nitrogen fixation, maintenance of a low redox potential, and prevention of entry of foreign microorganisms (Veivers et al. 1982; Charnley et al. 1985; Ohkuma et al. 1996; Leadbetter et al. 1999; Wenzel et al. 2002; Dillon et al. 2005; König 2006; Watanabe et al. 2006; Kudo 2009). However, relatively few studies have shown that Lepidoptera harbor gut bacteria. Similarly, few discuss the possibility that microorganisms may produce some of the digestive enzymes to provide essential nutrients or assist in important biochemical function related to host food ingestion (McKillip et al. 1997; Broderick et al. 2004; Xiang et al. 2006; Gao et al. 2007; Anand et al. 2010).

The silkworm, *Bombyx mori* L. (Lepidoptera: Bombycidae), as a model for Lepidoptera, is an agriculturally important insect for silk production. It mainly feeds on mulberry leaves, although tricuspid cudrania leaves can also act as the forage of the larvae (Li 1987). Recent studies have found that the silkworm larvae not only show retarded grow, but also are susceptible to *Bombyx mori* nucleopolyhedrovirus (BmNPV) when reared with tricuspid cudrania leaves. Compared with the mulberry leaves treatment group, the lethal dose 50 (LD_50_) of BmNPV (1.632 × 10^5^ PID/larva) is about one fourth in the tricuspid cudrania leaves group (Xue et al. 2009; Xie et al. 2009). In 2003, a study indicated the lipase isolated from silkworm larval alimentary canal shows strong antiviral activity against BmNPV, providing evidence that digestive juice may play an important role during peroral infection with BmNPV (Ponnuvel et al. 2003). Therefore, to explore the differences of intestinal bacteria between the BmNPV susceptible and non-susceptible silkworm, we used tricuspid cudrania leaves to feed silkworms in order to construct a BmNPV susceptible model. Furthermore, we investigated the dominant intestinal bacteria, namely the lipase-producing bacteria, and analyzed diversities and differences. This understanding of the diet-derived intestinal bacterial community may yield insight into relationships between gut bacteria and disease resistance of the silkworm.

## Materials and Methods

### Sample collection

Silkworm eggs were incubated using conventional methods. The silkworm larvae were reared with mulberry leaves from first to third instar, and then were divided into two identical groups; one reared with mulberry leaves, the other with tricuspid cudrania leaves, both at the start of the fourth instar. Twenty silkworm larvae of each treatment group were obtained every other day, from the first day of fourth instar to the seventh day of fifth instar, and subjected to starvation for 24 hours. Subsequently, the larvae were surface sterilized by submersion into 75% ethanol for one minute and rinsed three times using sterile distilled water prior to dissection. The larvae were dissected using dissection scissors and the guts were withdrawn with sterilized fine-tipped forceps. The intestinal juice of each five larvae was collected using sterile syringes and placed in a 5 mL sterilized centrifuge tube as one unit of collection for processing.

### Isolation of dominant bacteria

At the maximum dilution of intestinal juice, any bacterial colony that accounted for more than 10% the total colony was defined as a dominant microflora. Each sample collected could possess two or more types of dominant bacteria (Li 1999). According to the screening standard of dominant bacteria, 1 mL intestinal juice was added to 9 mL sterilized physiological saline, and tenfold serial dilution aliquots were used for the inoculation. On each agar plate, 0.1 mL of intestinal juice with maximum dilution was spread and incubated at 30 °C for 48–72 hours; all samples were repeated three times. The media used for the isolation of dominant bacteria included nutrient agar, potato dextrose agar, Gause's No. 1 agar, and Czaper's agar medium, which were autoclaved at 121 °C for 20 minutes, and pH value was adjusted to 9.2–9.8 (Huang 1999). Dominant colonies were picked out, purified three times by inoculating on the corresponding agar plates, and further transferred to agar slants.

### Identification of dominant bacteria

The dominant intestinal bacteria were identified by bacteriological properties and 16S rRNA gene sequencing. Morphological tests were done by standard procedures. The physiological-biochemical characteristics were determined on the basis of Gram stain, spore stain, oxidase test, catalase test, motility, Voges-Proskauer test, methyl red test, indole test, glucose fermentation test, hydrogen sulfide production, and hydrolysis of starch test (Dong and Cai 2001).

DNA was extracted by boiling bacterial cell suspension in sterile distilled water (Brousseau et al. 1993). 16S rRNA genes were amplified with universal primers of 27F (5'-GAGTTTGATCCTGGCTCAG-3') and 1492R (5'-CGGTTACCTTGTTACGACTT-3'). PCR amplification was performed in a total volume of 50 µL containing 5 µL DNA extract, 2 µL of 10 µmol/L of each primer, 4 µL of 5 mmol/L dNTPs, 4 µL of 25 mmol/L MgCl_2_, 5 µL of 10 × PCR buffer, and 0.3 µL of 5 U/µL Taq DNA polymerase using a thermal cycler. Cycling conditions were as follows: initial denaturation at 95 °C for five min, 29 cycles of 94 °C for one min, 55 °C for one min, 72 °C for one min, and a final extension at 72 °C for five min. PCR products were examined by electrophoresis in a 0.8% agarose gel, and bands were visualized by staining with ethidium bromide. PCR products were further purified with the QIAquick PCR purification kit (Quiagen, www.quiagen.com) and cloned into pMD18-T vector followed by sequencing. Sequence analysis was performed using the BLAST algorithm in GenBank (http://www.ncbi.nlm.nih.gov). Bacterial identifications were based on 16S rRNA gene sequence similarity. A phylogenetic tree was constructed with the neighbor-joining method (Saitou and Nei 1987) of MEGA 4.0 software using bootstrap values with 1000 replicates.

### Screening of lipase-producing bacteria

Each dominant isolate was inoculated on Rhodamine B agar plate medium (0.5% beef extract, 0.5% peptone, 0.5% (NH_4_)_2_SO_4_, 0.05% MgSO_4_, 0.4% K_2_HPO_4_, 0.02% CaCl_2_, 0.2% NaNO_3_, 12.0% olive oil emulsion, 2.0% Rhodamine B (0.1mg/mL), 1.8% agar, pH = 9.2–9.8) using a sterilized toothpick, and incubated at 30 °C for 48 hours. Lipase production was observed as a pink zone of hydrolysis around bacterial colony.

### Growth curve assay of lipase-producing bacteria

Each lipase-producing strain was inoculated in a 250 mL Erlenmeyer flask containing 25 mL of medium, consisting of 0.5% beef extract, 0.5% peptone, 0.5% sucrose, 0.3% NaCl, 0.2% K_2_HPO_4_, pH = 9.2–9.8, and incubated at 30 °C for 24 hours on a rotary shaker at 200 rpm. Subsequently, the inocula were transferred into production medium (2.0% peptone, 0.5% sucrose, 0.1% (NH_4_)_2_SO_4_, 0.1% MgSO_4_, 0.2% K_2_HPO_4_, 1.0% olive oil, pH = 9.2–9.8) at a rate of 2.0%, followed by incubation at 30 °C on a rotary shaker at 200 rpm. The samples were taken after every 12 hours until 72 hours. Cell density was measured by taking the optical density at 600 nm against the cell-free control.

### Assay of lipase activity

The production media were inoculated with 2.0% of seed cultures and incubated at 30 °C for 48 hours on a rotary shaker at 200 rpm. The cultures were centrifuged at 10,000 rpm for 10 min, and the supernatant was examined for lipase activity using an ultrasensitive lipase detection kit (Nanjing Jiancheng Bioengineering Institute, www.njjcbio.com/html_en/contact_us.php) by taking the optical density at 420 nm. One unit of lipase activity was defined as the amount of enzyme required for the consumption of 1 µmol substrate per minute under the standard assay condition.

**Table 1.**
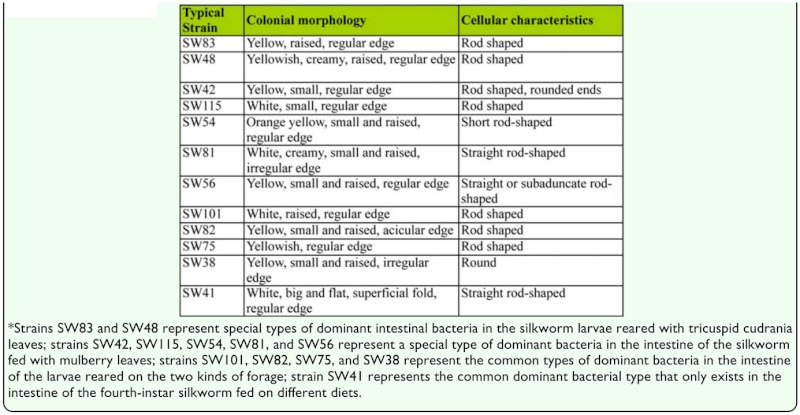
Morphological characteristics of dominant bacteria in the intestine of the silkworm larvae reared with mulberry leaves and tricuspid cudrania leaves.

## Results

### Isolation of dominant bacteria

Using the isolation procedure described above, a total of 56 dominant isolates were successfully collected from the intestine of the fourth and fifth instar silkworm larvae and classified into 12 phenotypes based on the colony color, size, and cellular morphology. Typical strains SW83, SW48, SW42, SW115, SW54, SW81, SW56, SW101, SW82, SW75, SW38, and SW41 represented the 12 phenotypes, respectively. Ten and seven phenotypes existed in the intestine of the larvae fed with mulberry leaves and tricuspid cudrania leaves, respectively; four of them were common in the intestine of both treatment groups (Table 1).

**Table 2.**
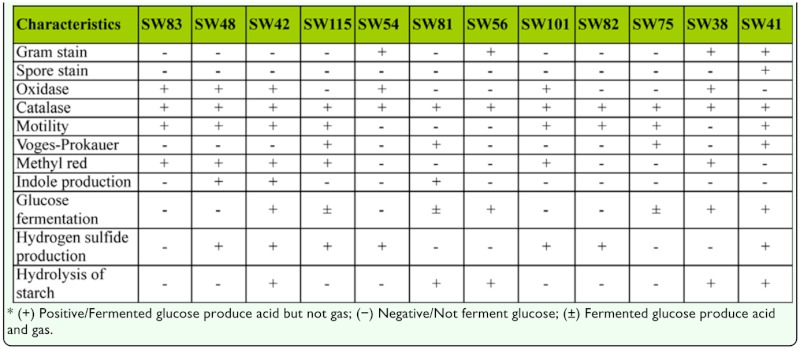
Physiological and biochemical characteristics of dominant bacteria in the silkworm intestine.

### Identification of dominant bacteria

Based on morphological and physiological-biochemical characteristics (Tables 1, 2), strains SW82 and SW83 were similar to Pseudomonadaceae, strains SW75, SW81, and SW115 were similar to Enterobacteriaceae, and strains SW48, SW42, SW54, SW56, SW101, SW38, and SW41 were similar to Rhizobiaceae, Vibrionaceae, Brevibacteriaceae, Corynebacteriaceae, Caulobacteraceae, Micrococcaceae, and Bacillaceae, respectively (Buchanan and Gibbons 1984).

**Figure 1.**
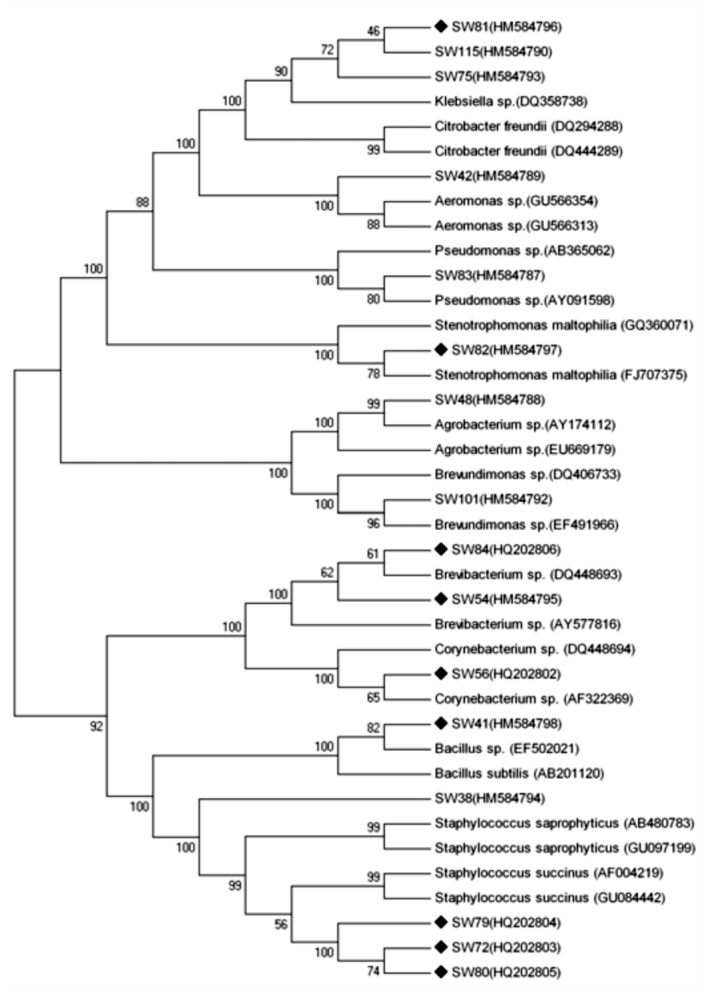
Phylogenetic tree of dominant bacteria in the intestine of the silkworm larvae reared with mulberry leaves and tricuspid cudrania leaves. The lipase-producing strains are labeled as black diamond. Number in parentheses represents the sequences accession number in GenBank. The numbers at each branch points indicate the percentage supported by bootstrap analysis based on 1000 resampled data sets. High quality figures are available online.

To further characterize the dominant intestinal bacteria, strain identification was performed using a molecular approach. The results showed that the 16S rRNA genes of all these isolates had ∼ 1500 bp DNA fragments by PCR amplification. All the sequences were deposited in the GenBank database. Accession numbers HM584787 to HM584790 corresponded to strains SW83, SW48, SW42, and SW115, accession numbers HM584792 to HM584798 corresponded to strains SW101, SW75, SW38, SW54, SW81, SW82, and SW41, and accession number HQ202802 corresponded to strain SW56. To clarify the phylogenetic position of these dominant bacteria, a phylogenetic tree was constructed based on 16S rRNA gene sequence homology (Figure 1). The results showed strains SW83, SW48, SW42, SW54, SW56, SW101, SW82, SW38, and SW41 had the highest homology with *Pseudomonas*, *Agrobacterium*, *Aeromonas*, Brevibacterium, *Corynebacterium*, *Brevundimonas*, *Stenotrophomonas*, *Staphylococcus*, and *Bacillus*, respectively. Strains SW115, SW81, and SW75 showed the highest homology with *Klebsiella.* However, together with the morphological and physiological-biochemical characteristics, strains SW115, SW81, and SW75 belonged to *Citrobacter*, *Klebsiella*, and *Enterobacter*, respectively.

### Screening of lipase-producing bacteria

**Figure 2.**
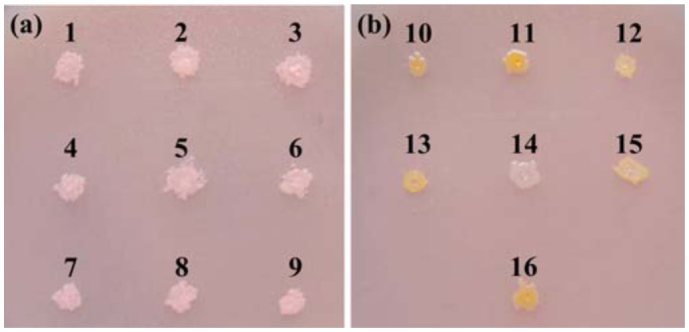
Results of screening the dominant strains on Rhodamine B agar plates, (a) Pink zones 1–9 indicate the hydrolytic zones of lipase-producing strains SW41, SW54, SW56, SW72, SW79, SW80, SW81, SW84, and SW82, respectively. (b) Colonies 10–15 represent bacterial types SW48 (*Agrobacterium*), SW83 (*Pseudomonas*), SW73 (*Bacillus*), SW38 (*Staphylococcus*), SW101 (*Brevundimonas*), and SW75 (*Enterobacter*), which do not producing lipase in the intestine of the silkworm larvae reared with tricuspid cudrania leaves; colony 16 represents bacterial type SW42 (*Aeromonas*) which do not producing lipase in the intestine of the larvae reared with mulberry leaves. High quality figures are available online.

According to screening the isolates on Rhodamine B agar plate, nine lipase-producing bacteria were obtained and named as SW41, SW54, SW56, SW72, SW79, SW80, SW81, SW84, and SW82, respectively (Figure 2). Except for strain SW82, which was common in both, the other bacteria only existed in the intestine of the silkworm larvae reared with mulberry leaves.

As identified above, strains SW41, SW54, SW56, SW81, and SW82 were *Bacillus, Brevibacterium, Corynebacterium, Klebsiella, and Stenotrophomonas*, respectively. 16S rRNA gene sequencing further identified the other lipase-producing bacteria. Strains SW72, SW79, and SW80 (accession numbers HQ202803 to HQ202805) showed the highest similarity to *Staphylococcus*, and strain SW84 with accession number HQ202806 showed the highest similarity to *Brevibacterium* (Figure 1).

### Growth curve assay of lipase-producing bacteria

**Figure 3.**
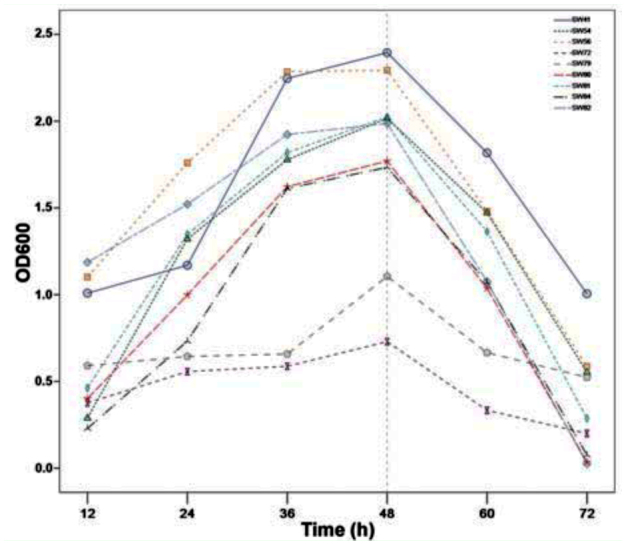
Growth curve of lipase-producing bacteria in the intestine of the silkworm larvae reared with mulberry leaves and tricuspid cudrania leaves. High quality figures are available online.

The bacterial density was examined after every 12 hours until 72 hours; the maximum cell density of all the lipase-producing bacteria was observed at ∼ 48 hours of fermentation. After that, the bacterial density reduced, probably due to the consumption of nutrients. The maximum bacterial density showed a significant difference; strain SW41 had the maximum yield, whereas strain SW72 had the minimum yield (Figure 3).

### Assay of lipase activity

**Figure 4.**
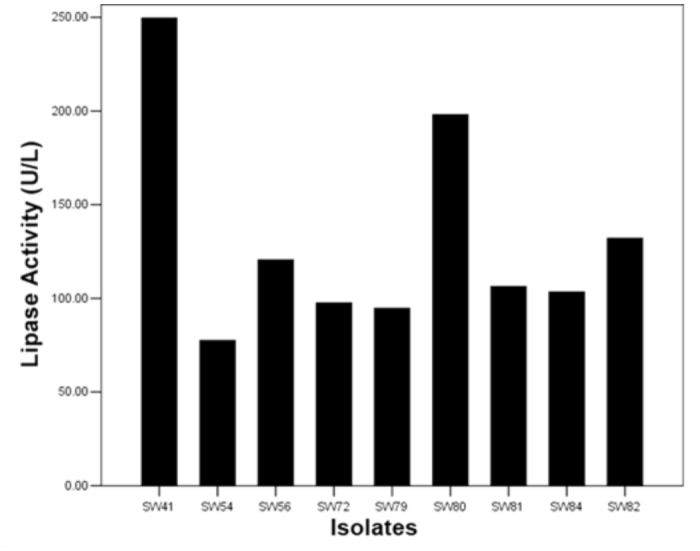
Lipase activity of lipase-producing bacteria in the intestine of the silkworm larvae reared with mulberry leaves and tricuspid cudrania leaves. High quality figures are available online.

Lipase activity was determined using a SmartSpecTM3000 UV spectrophotometer (Bio-Rad, www.bio-rad.com). Results showed the maximum total lipase activity after 48 hours of fermentation *in vitro.* At their maximum bacterial density, strain SW41 showed the highest lipase activity (249.59 U/L), while strain SW54 showed the lowest activity (77.46 U/L) (Figure 4).

## Discussion

This study showed that four phylotypes of the isolated dominant bacteria are common in the intestine of both the silkworm larvae fed on different forage, including *Brevundimonas*, *Stenotrophomonas*, *Enterobacter*, and *Staphylococcus.* There are five phylotypes including *Aeromonas*, *Citrobacter*, *Brevibacterium*, *Klebsiella* and *Corynebacterium*, which only exist in the intestine of the larvae reared with mulberry leaves, while the larvae fed with tricuspid cudrania leaves possess two different genera, *Pseudomonas* and *Agrobacterium.* Based on the isolated dominant strains, nine lipase-producing bacteria were obtained and classified into six genera including *Bacillus*, *Brevibacterium*, *Corynebacterium*, *Staphylococcus*, *Klebsiella*, and *Stenotrophomonas.* The common bacterium belongs to genus *Stenotrophomonas*, and the other bacteria only exist in the intestine of the silkworm larvae reared with mulberry leaves. The diversity of lipase-producing bacteria from the intestine of the silkworm feeding on tricuspid cudrania leaves was considerably deficient compared with that of the larvae reared with mulberry leaves. This difference may relate to the different content of lipids. Additionally, tricuspid cudrania leaves contain fewer sterols than that of mulberry leaves; sterols are indispensable in growth and development of the silkworm larvae (Xue 2009). The lipase-producing bacterial community likely shows great changes in response to the food source. These results provide evidence that diet has a significant impact on gut microbial community. This appears to the case in other lepidopteran caterpillars such as the gypsy moth, with 15 phylotypes at its most complex and 7 phylotypes at its simplest (Broderick et al. 2004). Diet is also known to influence the intestinal microorganism community in cockroaches and crickets, where microbial populations fluctuate in response to dietary changes (Kane and Breznak 1991; Santo Domingo et al. 1998).

Based on microecology theory, insects lack a complete enzyme system and thus need gut microorganisms to provide different kinds of enzymes for food digestion, nutrient absorption, and biological metabolism (Wei 1985). In this study, the pH value of the media for lipase-producing bacteria was nearly 10.0, which is similar to the gut pH of the silkworm. It suggested that the isolates were able to produce lipase in a similar gut environment. This suggests that not all gut lipase is secreted by the silkworm cells; a recent study reported that a part of velvetbean caterpillar gut protease was secreted by their gut bacteria (Visôtto et al. 2009). Although the maximum bacterial density and lipase activity were examined at ∼ 48 hours *in vitro*, the properties of these bacteria still need to be studied *in vivo.* However, the understanding of microecology in BmNPV susceptible and non-susceptible silkworms is the basis of disclosing the relationship between intestinal bacteria and BmNPV infection. This study suggests that lipase secreted by gut bacteria might be related to BmNPV resistance in the silkworm.

## Abbreviations:

**BmNPV**,: *Bombyx mori* nucleopolyhedrovirus
